# Functional Expression of Choline Transporters in Microglia and Their Regulation of Microglial M1/M2 Polarization

**DOI:** 10.3390/ijms23168924

**Published:** 2022-08-10

**Authors:** Toshio Okada, Eisuke Muto, Tsuyoshi Yamanaka, Hiroyuki Uchino, Masato Inazu

**Affiliations:** 1 Department of Anesthesiology, Tokyo Medical University, 6-7-1 Nishishinjuku, Shinjuku-ku, Tokyo 160-0023, Japan; 2 Department of Molecular Preventive Medicine, Tokyo Medical University, 6-1-1 Shinjuku, Shinjuku-ku, Tokyo 160-8402, Japan; 3 Institute of Medical Science, Tokyo Medical University, 6-1-1 Shinjuku, Shinjuku-ku, Tokyo 160-8402, Japan

**Keywords:** microglia, choline transporter, M1/M2 polarization

## Abstract

Background: Microglia are key cells of the immune system in the central nervous system and are suggested to be deeply involved in the development of neurodegenerative diseases. It is well known that microglia have functional plasticity, with an inflammatory M1 phenotype and an anti-inflammatory M2 phenotype. Inhibition of choline transport in macrophages has been reported to suppress the secretion of inflammatory cytokines. However, the role of the choline transport system in regulating microglial M1/M2 polarization has not been fully elucidated to date. In this study, we investigated the mechanism of choline uptake in microglia, and its association with microglial M1/M2 polarization. Methods: The immortalized mouse microglial cell line SIM-A9 was used for [^3^H]choline uptake and expression analysis of choline transporters. The association between the choline uptake system and the M1/M2 polarization of microglia was also analyzed. Results: Choline transporter-like protein (CTL) 1 and CTL2 were highly expressed in SIM-A9 cells, and CTL1 and CTL2 were localized in the plasma membrane and mitochondria, respectively. Functional analysis of choline uptake demonstrated the existence of Na^+^-independent, pH-dependent, and intermediate-affinity choline transport systems. Choline uptake was concentration-dependently inhibited by hemicholinium-3 (HC-3), an inhibitor of choline uptake, and increased by lipopolysaccharide (LPS) and interleukin-4 (IL-4). Expression of the mRNA of M1 microglia markers IL-1β and IL-6 was increased by LPS, and their effects were suppressed by choline deprivation and HC-3. In contrast, mRNA expression of the M2 microglial marker arginase-1 (Arg-1) was increased by IL-4, and the effect was enhanced by choline deprivation and HC-3. Conclusions: Our results suggest that inhibition of CTL1-mediated choline uptake in microglia preferentially induces M2 microglia polarization, which is a potential therapeutic approach for inflammatory brain diseases.

## 1. Introduction

Glial cells represent all non-neuronal cells in the central nervous system (CNS) and comprise more than 90% of cell in the human brain. They are composed of two major populations: macroglia, consisting of astrocytes and oligodendrocytes, and microglia [1]. Microglia are myeloid cells derived from yolk sac progenitors of the CNS and are observed in the brain very early in embryonic life [2,3,4]. Microglia have long been recognized as endogenous innate immune cells present in the CNS. They not only perform local immune-associated functions, but also provide crosstalk between the CNS and the entire immune system. In the adult CNS, microglia are involved in brain homeostasis, regulation of synaptic transmission, and clearance of apoptotic cells. During embryogenesis, microglia are involved in the removal of excess synapses and neurons, and in the formation of neuronal networks [5]. They are of mesodermal/mesenchymal origin, migrate to all areas of the CNS, seed the brain parenchyma, and acquire a unique ramified morphological phenotype called “resting microglia” [6]. Microglia exist in a resting state in the healthy brain. Various stimuli can rapidly “activate” these resting microglia, and activated microglia are implicated in many neurodegenerative disease processes, including those of Alzheimer’s disease, Parkinson’s disease, and amyotrophic lateral sclerosis. Activated microglia are classified into two opposing types with different phenotypes: the M1 phenotype and the M2 phenotype. M1 microglia respond to injury and infection, generally defending tissues in the first step and facilitating the destruction of invading pathogens in the second step. However, they also induce neurotoxicity by releasing various neurotoxic mediators and proinflammatory factors, often leading to a vicious cycle of neuronal death and acute inflammation. M2 microglia are the primary effector cells that can dampen inflammatory immune responses and promote the expression of repair genes [7]. Macrophages are known to release inflammatory cytokines upon lipopolysaccharide (LPS) stimulation, which is a change similar to the M1 polarization of activated microglia. Previous reports have shown that LPS-stimulated macrophage stimulation rapidly increases phosphatidylcholine (PC) synthesis [8,9]. It is also known that when PC synthesis is inhibited by a deficiency of bone marrow-specific cytidyltransferase (CCT: the rate-limiting enzyme for PC synthesis), the ability of macrophages to secrete inflammatory cytokines in response to LPS is reduced [10]. These results suggest that choline availability and PC synthesis may be important for macrophage activation associated with inflammation.

Choline is a constituent of PC and sphingomyelin, which are the main components of cell membranes, and are essential biofactors in all cells. The choline to PC pathway (called the Kennedy pathway) involves three synthetic enzymes: choline kinase, CTP phosphocholine cytidylyltransferase, and choline phosphotransferase. Choline also functions as a biochemical precursor to the neurotransmitter acetylcholine (ACh), the lipid mediator platelet-activating factor, and the methyl group donor betaine. Thus, after being taken up into cells, choline is metabolized into molecules necessary for living organisms, and is involved in various physiological actions. Therefore, choline must be taken up into cells to be utilized. Under physiological conditions, choline exists as a quaternary ammonium ion with a positive charge, so a carrier protein is present to transport it across the cell membrane [11]. Prior to identification of the choline transporter molecule, the existence and the properties of two types of choline transport systems had been reported [12,13]. The first is a Na^+^-dependent, high-affinity transport system with the kinetics parameters Michaelis constant (*K_m_* value) of 0.5 to 3 μM for choline, and is sensitive to low concentrations of hemicholinium-3 (HC-3), which is a choline analog and a choline uptake inhibitor. This transport system is associated with the acetylcholine synthesis system and is found in the nervous system. The second is a Na^+^-independent, low-affinity transport system with a *K_m_* value of 10 to 60 μM for choline, is sensitive to high concentrations of HC-3, is found in many cells, and is thought to be associated with the phospholipid synthesis system. The choline transport system is currently classified into three families of transporters: high-affinity choline transporter 1 (CHT1/SLC5A7), choline transporter-like proteins (CTL1-5/SLC44A1-5), and polyspecific organic cation transporters (OCT1-3/SLC22A1-3). Each transporter has different characteristics, including affinity for tissue-distributed choline, sensitivity to HC-3, Na^+^-dependence, and substrate specificity [14].

Recently, the M1/M2 polarity of microglial activation in several neurodegenerative diseases has been studied to elucidate the mechanisms of their immunopathology, but the link to choline has not been clarified. Therefore, the aim of this study was to investigate the effects of choline transporter expression and function on this microglia phenotype switch. We used the mouse microglia cell line, SIM-A9 in our experiments. LPS promotes M1 polarization of microglia and inflammatory cytokine secretion [15]. Interleukin-4 (IL-4) promote M2 polarization of microglia and macrophages via the JAK1/STAT6 pathway [16]. Therefore, LPS and IL-4 were used to induce M1/M2 polarization in this experiment. Our study focused on the effects of choline deficiency and inhibition of choline transporter activity on each type of activated microglia.

## 2. Results

### 2.1. Expression of Choline Transporters in SIM-A9 Cells

**Figure 1:**
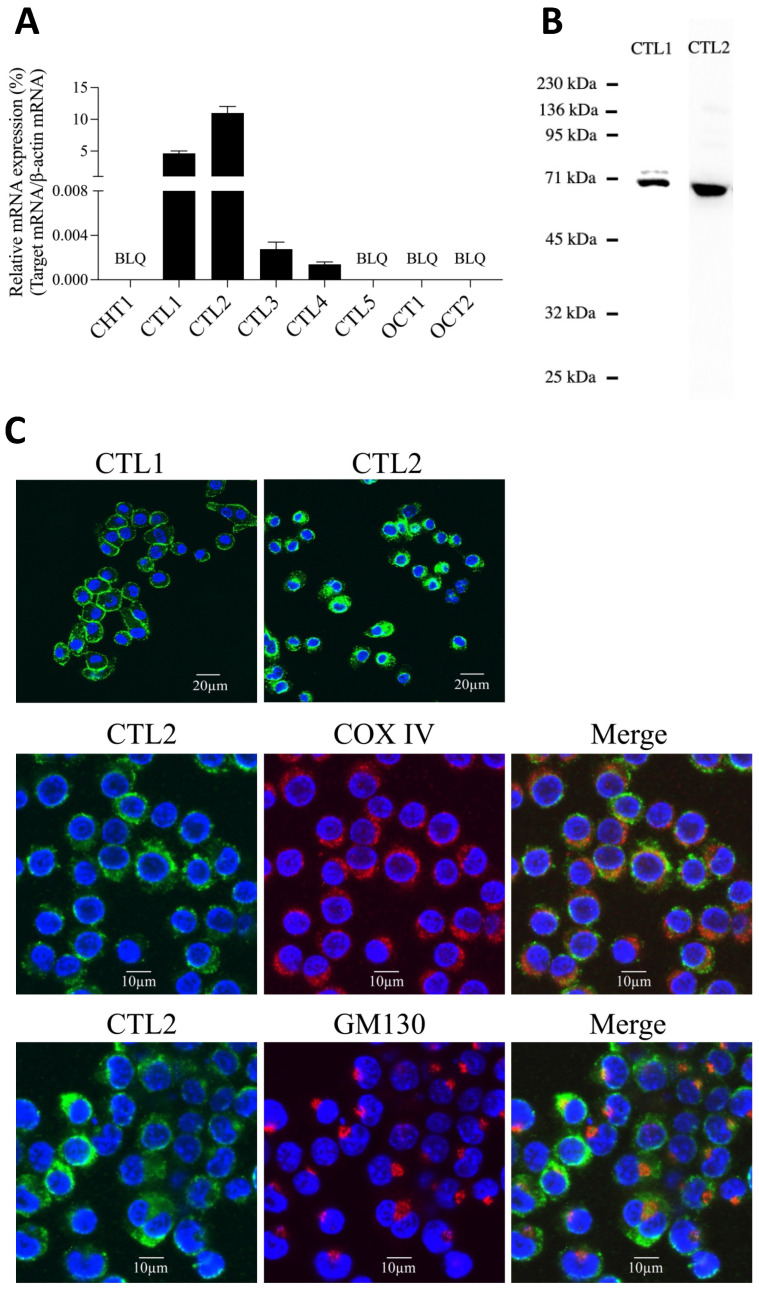
Expression of choline transporters in SIM-A9 cells. (**A**) Expression of the mRNA of choline transporters CHT1, CTL1-5, and OCT1-2. Real-time PCR was used to analyze the mRNA expression of choline transporters, and the results are expressed as the ratio of target mRNA to β-actin mRNA. Data are shown as the mean ± SD (*n* = 3). BLQ, below the limit of quantification. (**B**) Detection of CTL1 and CTL2 proteins by Western blot analysis. (**C**) Subcellular distribution of CTL1 and CTL2 proteins was analyzed by immunocytochemistry followed by confocal microscopy. CTL1 and CTL2 are shown in green, and MG130 and COV IV in red. Nuclei were stained with DAPI (blue). Merged images indicate colocalization.

We first analyzed the mRNA expression of CHT1, CTL1-5, and OCT1-2 in SIM-A9 cells by real-time polymerase chain reaction (PCR). In SIM-A9 cells, CTL1 and CTL2 mRNA were expressed at high levels, whereas CHT1, CTL5, OCT1, and OCT2 mRNA were below the detection limit. In addition, CTL3 and CTL4 mRNA were expressed at very low levels (Figure 1A). As CTL1 and CTL2 mRNA were expressed at high levels, their protein expression levels were analyzed by Western blot analysis. Immunoblotting with CTL1 and CTL2 antibodies detected bands of 70 kDa and 60 kDa, respectively, confirming the expression of both CTL1 and CTL2 proteins (Figure 1B). The subcellular distribution of CTL1 and CTL2 in SIM-A9 cells was determined using immunofluorescent staining. CTL1 immunoreactivity was identified primarily at the cell surface (Figure 1C). On the other hand, immunoreactivity of CTL2 was confirmed intracellularly, overlapping with the immunoreactivity of the mitochondrial marker, anti-cytochrome c oxidase IV (COX IV), but not with GM130, a marker of the Golgi apparatus (Figure 1C). Thus, CTL1 and CTL2 mRNA and protein were expressed in SIM-A9 cells, with CTL1 localized to the plasma membrane and CTL2 to mitochondria.

### 2.2. Properties of [^3^H]Choline Uptake in SIM-A9 Cells

**Figure 2:**
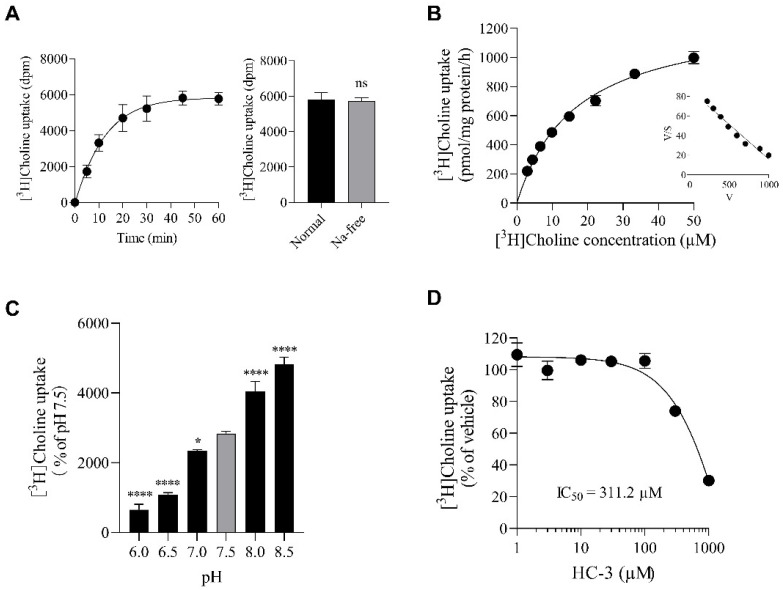
Characterization of [^3^H]choline uptake in SIM-A9 cells. (**A**) Time course and Na^+^ dependence of 10 μM [^3^H]choline uptake. [^3^H]Choline uptake in the presence and absence of Na^+^ for 45 min was not significantly different. ns: no significant difference. Data were analyzed with the unpaired *t*-test. (**B**) Kinetics of [^3^H]choline uptake. Michaelis–Menten kinetics of [^3^H]choline uptake demonstrated a *K_m_* of 16.5 μM and a *V_max_* of 1303.0 pmol/mg protein/h. Each point represents the mean ± S.D. (*n* = 4). Inset: Eadie–Hofstee plot of [^3^H]choline uptake showing a single straight line. (**C**) Effects of extracellular pH (pH6 to 8.5) on choline uptake. [^3^H]Choline uptake at each extracellular pH was expressed as the percentage of choline uptake at pH 7.5. Each column represents the mean ± SD (n = 4). * *p* < 0.05 and **** *p* < 0.0001 compared with pH 7.5. Data were analyzed with the Dunnett’s multiple comparisons test. (**D**) Effects of the choline uptake inhibitor HC-3 on [^3^H]choline uptake. SIM-A9 cells were preincubated for 20 min with various concentrations of HC-3, and then their uptake of 10 µM [^3^H]choline was followed for 20 min. The IC_50_ value of inhibition of [^3^H]choline uptake by HC-3 in SIM-A9 was 311.2 μM. Results are shown as the percentage of the control uptake, measured in the presence of the vehicle. Each point represents the mean ± SD (*n* = 3).

We investigated the effect of extracellular Na^+^ on the time course of [^3^H]choline uptake in SIM-A9 cells (Figure 2A). [^3^H]Choline uptake increased in a time-dependent manner, and was linear with time until at least 10 min. When NaCl in the uptake buffer was replaced with an equimolar amount of N-methyl-D-glutamine chloride (NMDG-Cl), there was no difference in the uptake of [^3^H]choline under Na^+^-free conditions at 45 min. These results indicate that choline uptake in SIM-A9 cells occurs via a Na^+^-independent transport system.

We characterized the kinetics of [^3^H]choline uptake by SIM-A9 cells (Figure 2B). Kinetic analysis of [^3^H]choline uptake calculated by nonlinear regression analysis showed a *K_m_* value of 16.5 μM and a *V_max_* of 1303 pmol/mg protein/h, and Eadie–Hofstee plots showed a straight line. These data suggest that choline uptake in SIM-A9 cells is mediated by a single choline transport system with intermediate affinity.

In various cultured cells, CTL1-mediated choline uptake has been reported to be pH-dependent [17,18,19]. We hence investigated [^3^H]choline uptake in SIM-A9 cells at different extracellular pHs (pH 5.5 to 8.5) (Figure 2C). Acidification of the extracellular pH (pH 7.5 to 5.5) significantly decreased [^3^H]choline uptake. Conversely, increasing the extracellular pH to alkaline (pH 7.5 to 8.5) significantly increased [^3^H]choline uptake. 

Next, the inhibitory effects of the choline uptake inhibitor HC-3 on the uptake of [^3^H]choline in SIM-A9 cells was analyzed (Figure 2D). HC-3 inhibited [^3^H]choline uptake in a concentration-dependent manner, with an IC_50_ value of 311.2 μM.

### 2.3. Effects of LPS and IL-4 on [^3^H]Choline Uptake in SIM-A9 Cells

**Figure 3:**
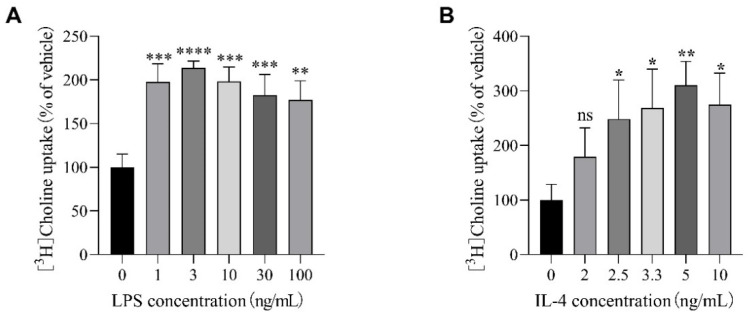
Effects of LPS and IL-4 on [^3^H]choline uptake in SIM-A9 cells. Cells were preincubated with various concentrations of LPS (**A**) or IL-4 (**B**) for 5 h, and the uptake of 10 µM [^3^H]choline was then measured for 20 min. Results are shown as the percentage of the control uptake, measured in the presence of vehicle. Each column represents the mean ± S.D. (*n* = 4). * *p* < 0.05, ** *p* < 0.01, *** *p* < 0.001, and **** *p* < 0.0001 compared with vehicle. Data were analyzed with the Dunnett’s multiple comparisons test. ns: no significant difference.

We next analyzed the effects of LPS and IL-4 on [^3^H]choline uptake in SIM-A9 cells. Cells were preincubated with various concentrations of LPS or IL-4 for 5 h, and the uptake of 10 µM [^3^H]choline at 20 min was measured. LPS treatment significantly increased [^3^H]choline uptake at all concentrations (Figure 3A). IL-4 administration showed a slight increase at 2 ng/mL, although not statistically significant, and significantly increases at all higher concentrations (Figure 3B).

### 2.4. Effects of Choline Deficiency on IL-1β, IL-6, and Arg-1 mRNA Expression in SIM-A9 Cells Stimulated with LPS and IL-4

**Figure 4:**
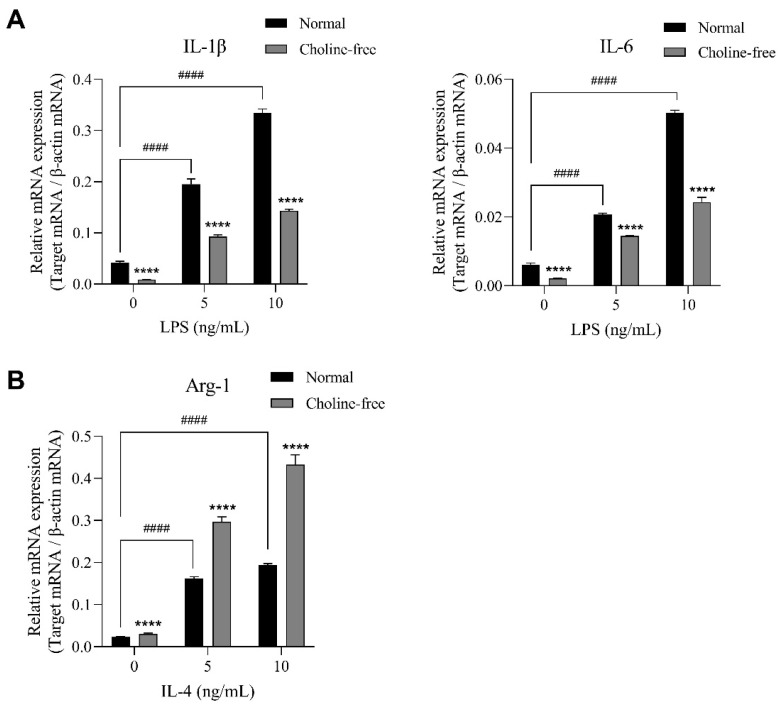
Effects of choline deficiency on IL-1β, IL-6, and Arg-1 mRNA expression in SIM-A9 cells stimulated with LPS or IL-4. (**A**) Expression of IL-1β and IL-6 mRNA in M1 microglia stimulated with LPS for 4 h in normal medium or choline-free medium. (**B**) Expression of Arg-1 mRNA in M2 microglia stimulated with IL-4 for 4 h in normal medium or choline-free medium. Relative expression is expressed as a ratio of the target mRNA to β-actin mRNA. Data are presented as the mean ± SD (*n* = 3). **** *p* < 0.0001 compared with normal medium. Data were analyzed with the Šídák’s multiple comparisons test. #### *p* < 0.0001 compared with vehicle control. Data were analyzed with the Dunnett’s multiple comparisons test.

We analyzed the mRNA expression levels of the M1 microglial markers IL-1β and IL-6, and the M2 microglial marker Arginase-1 (Arg-1) in SIM-A9 cells stimulated with LPS or IL-4. SIM-A9 cells were stimulated with various concentrations (0, 5, and 10 ng/mL) of LPS or IL-4 for 4 h in the presence of normal medium and choline-free medium. LPS-stimulated SIM-A9 cells showed significantly increased mRNA levels of IL-1β and IL-6 in a concentration-dependent manner in normal medium. These increasing effects were significantly suppressed by choline deficiency (Figure 4A). IL-4-stimulated SIM-A9 cells also showed significantly increased mRNA levels of Arg-1 in a concentration-dependent manner in normal medium. However, these increasing effects were significantly enhanced by choline deficiency (Figure 4B).

### 2.5. Effect of HC-3 on IL-1β, IL-6, and Arg-1 mRNA Expression in SIM-A9 Cells Stimulated by LPS and IL-4

**Figure 5:**
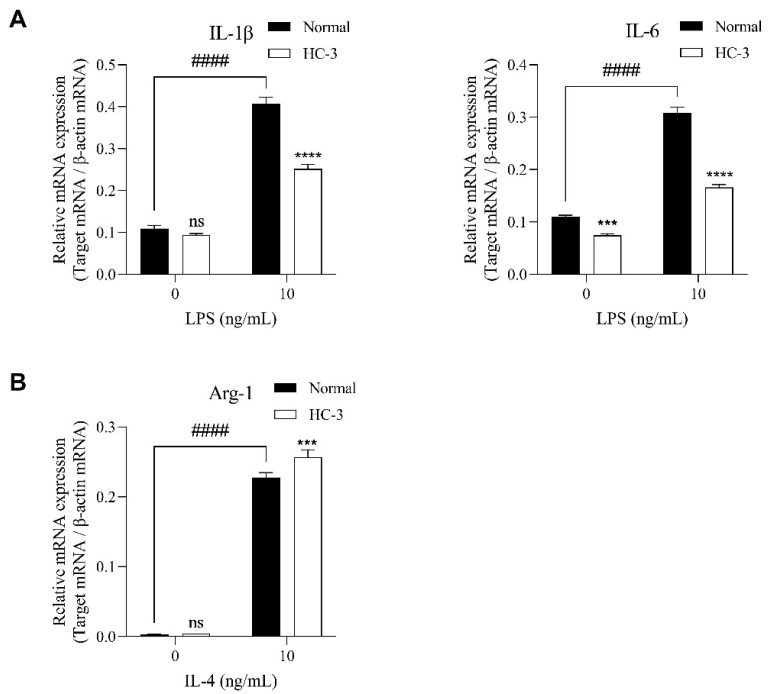
Effects of HC-3 on IL-1β, IL-6, and Arg-1 mRNA expression in SIM-A9 cells stimulated with LPS or IL-4. (**A**) Expression of IL-1β and IL-6 mRNA in M1 microglia stimulated with LPS for 4 h in the presence and absence of 1 mM HC-3. (**B**) Expression of Arg-1 mRNA in M2 microglia stimulated with IL-4 for 4 h in the presence and absence of 1 mM HC-3. Relative expression is shown as the ratio of target mRNA to β-actin mRNA. Data are presented as the mean ± SD (*n* = 3). *** *p* < 0.001, **** *p* < 0.0001 compared with normal medium. Data were analyzed with the Šídák’s multiple comparisons test. #### *p* < 0.0001 compared with vehicle control. Data were analyzed with the unpaired *t*-test. ns: no significant difference.

Lastly, we analyzed the effects of the choline uptake inhibitor HC-3 on IL-1β, IL-6, and Arg-1 mRNA expression in SIM-A9 cells stimulated with LPS and IL-4. LPS (10 ng/mL)-stimulated SIM-A9 cells showed significantly increased mRNA levels of IL-1β and IL-6 compared with control vehicle-treated cells. These increasing effects were significantly suppressed by 1 mM HC-3 treatment (Figure 5A). IL-4 (10 ng/mL)-stimulated SIM-A9 cells also showed significantly increased mRNA levels of Arg-1 compared with control vehicle-treated cells. However, these increasing effects were significantly enhanced by 1 mM HC-3 treatment (Figure 5B).

## 3. Discussion

Choline is a biofactor that plays an important role in the body, and is a synthetic material for phospholipids, such as phosphatidylcholine (PC) and sphingomyelin. Extracellular choline uptake via the choline transporter is the rate-limiting step in the synthesis of phospholipid, which is a major component of cell membranes. It has been suggested that Toll-like receptor (TLR) activation by LPS enhances CTL1-mediated choline uptake in macrophages [20]. However, the association between microglial activation and choline uptake has not been fully elucidated. Therefore, in this study, we first investigated the expression pattern and function of the choline transporters expressed in mouse microglia SIM-A9 cells. Analysis of the mRNA expression levels of choline transporters demonstrated that CTL1 and CTL2 were highly expressed, and their expression at the protein level was also confirmed. In addition, immunocytochemistry revealed that CTL1 was clearly present in the plasma membrane and CTL2 was predominantly present in the mitochondria. Thus, CTL1 may be involved in the transport of extracellular choline.

Therefore, we next performed functional analysis of choline uptake in SIM-A9 cells. We found that [^3^H]choline uptake in SIM-A9 cells is mediated by a single uptake mechanism that is Na^+^-independent, pH-dependent, and of intermediate affinity (*K_m_* = 16.5 µM). In a previous report, CTL2 had a lower affinity than CTL1, with a *K_m_* value of 210.6 μM in JEG-3 cells [21]. These biochemical findings lead us to conclude that choline uptake in SIM-A9 cells is mediated by CTL1, not CTL2. These properties of choline uptake in SIM-A9 cells are similar to those previously reported for CTL1 [22,23]. The *K_m_* value of choline uptake in SIM-A9 cells was 16.5 μM, which was close to the concentration of choline in the cerebrospinal fluid of healthy adults [24]. It is presumed that CTL1 is primarily responsible for choline uptake in the physiological brain environment. Pharmacological analysis using the choline uptake inhibitor HC-3 showed that HC-3 inhibits CTL1-mediated choline uptake in the μM range. In contrast, CHT1-mediated choline uptake is inhibited in the nM range [11,23]. The IC_50_ value of HC-3 was 311.2 μM, suggesting that CTL1 is functionally expressed in SIM-A9 cells. These biochemical and pharmacological analyses demonstrated that extracellular choline uptake in SIM-A9 cells is mediated by CTL1.

Extensive neuroinflammation is thought to play a role in the development and progression of neurodegenerative diseases, such as Parkinson’s disease and Alzheimer’s disease [25]. In general, microglia in the brain are known to exist in two states, M1 microglia and M2 microglia. Under stress, M1 microglia are induced and activated, releasing inflammatory factors and triggering neuroinflammatory responses. After inflammation subsides, microglia transform into differently activated M2 microglia, which play a neuroprotective role [26]. LPS, which is an endotoxin, activates innate immune responses through Toll-like receptor 4 (TLR4) and its core receptor, myeloid differentiation factor [27]. IL-4 is a well-known factor that promotes M2 polarization in microglia and macrophages. It is a multifunctional cytokine secreted primarily by Th2 cells, mast cells, eosinophils, basophils, and stromal cells [28]. It is well known that IL-4 regulates a variety of immune responses, including T cell differentiation and B cell Immunoglobulin E class switching. Serum IL-4 levels are also known to increase significantly in patients several hours after a stroke [29]. As IL-4 deficiency was shown to exacerbate brain damage and worsen neurological outcome 24 h after transient middle cerebral artery occlusion in animal stroke models [30,31], IL-4 is thought to function as an endogenous neuroprotective molecule immediately after stroke onset. Thus, several lines of evidence indicate that IL-4 plays an important role in brain function under physiological and pathological conditions. In the present study, LPS and IL-4 were used to induce mouse microglia SIM-A9 to change into the M1 or M2 types, respectively, similarly to in previous reports [7,15,26,32]. In addition, [^3^H]choline uptake in SIM-A9 cells was enhanced by stimulation with LPS and IL-4. This may also be due to the enhanced uptake of choline, which contributes to PC synthesis, in microglial activation. Previous studies have shown that choline is taken up by macrophages in pathological inflammatory conditions [33]. Recent studies have shown that TLR4-mediated macrophage activation induces the nuclear factor κB-dependent choline transporter CTL1, which upregulates choline uptake, and the newly taken-up choline is rapidly converted to PC via the Kennedy pathway [20]. Suppression of CTL1 expression and choline metabolism alter mitochondrial phospholipid composition, leading to the accumulation of defective mitochondria that are rapidly removed by mitophagy [20]. These results strongly suggest that choline uptake is responsible for phospholipid remodeling and maintenance of mitochondrial function in macrophage metabolism. Choline cytidyltransferase (CCT) is the rate-limiting enzyme in the PC biosynthetic pathway. PC synthesis by CCT is required for the normal structure and function of the Golgi apparatus in macrophages and for the maintenance of cytokine secretion from the Golgi apparatus, it has been reported that tumor necrosis factor-α and IL-6 secretion from the Golgi is inhibited in macrophages lacking CCTα [10]. In our study, choline deficiency and CTL1 inhibition both suppressed the increased mRNA levels of pro-inflammatory cytokine IL-1β and IL-6 by LPS stimulation in SIM-A9 cells. This suggests that PC production in the Kennedy pathway in choline metabolism plays an important role in inflammatory cytokine secretion in microglia as well as in macrophages. Therefore, decreased production of PC means that secretion of cytokine vesicles from the Golgi are inhibited.

The results of our present study show that choline deficiency and CTL1 inhibition suppressed cytokine secretion in M1 microglia, but enhanced Arg-1 secretion in M2 microglia. In microglia of mice after cerebral infarction, M2-like responses show a transient increasing trend in the first 1 to 2 days, then the phenotype switches from M2-like to M1-like, with a decrease in M2-like responses and a peak in M1-like responses on day 14 [34,35,36]. Furthermore, in microglia of mice after cerebral hemorrhage, M1-like responses are increased at 6 h after hemorrhage, and M2-like responses begin to increase from the first day after hemorrhage; a mixed M1 and M2 microglial phenotype is seen from day 1 to 3, with evidence supporting a switch in phenotype from M1 to M2 within the first 7 days [37,38,39]. These results suggest that microglia in injured tissues demonstrate either M1 or M2 polarity over time, in which an increase in the characteristics of one type leads to a decrease in the characteristics of the other. Our results demonstrated that choline deficiency or inhibition of choline uptake decreased M1 marker cytokines, which may have resulted in a secondary increase in M2 microglia. Choline deprivation or inhibition of choline synthesis enhanced the upregulating effects of M2 markers in activated microglia. This may enhance neuroprotection and potentially treat inflammatory diseases of the CNS by promoting a change in polarity from M1 to M2 microglia, which has been the focus of much attention in recent years.

Although we present novel findings involving CTL1 function in M1/M2 switching in microglia, a major limitation of this study is that only SIM-A9 cells were used. In the future, it is necessary to carry out experiments using primary microglia and the role of choline transporters on microglia polarization in animal models such as Alzheimer’s disease with brain inflammation.

In conclusion, CTL1 is functionally expressed in microglia and is responsible for extracellular choline uptake. Inhibition of the CTL1-mediated uptake of choline also switches the polarity from M1 to M2 microglia. CTL1 expressed in microglia may be a therapeutic target molecule for inflammatory neuropathies.

## 4. Materials and Methods

### 4.1. Materials

LPS-B5 purchased from Invitrogen Inc. (Sorrento Valley Blvd, San Diego, CA, USA), was used as the M1 stimulator in this experiment. LPS-B5 is highly pyrogenic and primarily a potent activator of TLR4 with the subsequent induction of NF-kB and the production of pro-inflammatory cytokines [40]. IL-4 was purchased from Peprotech Inc (Crescent Ave, Rocky Hill, NJ, USA). HC-3 was purchased from Sigma-Aldrich (St. Louis, MO, USA). [methyl-^3^H]Choline chloride (specific activity: 2800 GBq/mmol) was obtained from PerkinElmer Life Sciences, Inc. (Waltham, MA, USA).

### 4.2. Cell Culture

The immortalized mouse microglia cell line, SIM-A9, was purchased from Applied Biological Materials Inc. (Richmond, Canada). These cells express the macrophage/microglia-specific proteins CD68 and Iba1 and can be induced to pro- or anti-inflammatory M1/M2 microglial phenotypes [41]. Cells were grown in RPMI 1640 medium (Wako, Osaka, Japan) supplemented with 10% fetal bovine serum (Gibco, Grand Island, NY, USA) and Penicillin-Streptomycin Solution (Wako) in non-coated flasks and 24-well plates. Cells were cultured at 37 °C in a humidified atmosphere of 5% CO_2_ and 95% air, and the medium was changed every 3 to 4 days.

### 4.3. RNA Extraction and Real-Time PCR Assay

Levels of mRNA expression were measured according to previously established methods [19,42,43]. Total RNA was extracted from SIM-A9 cells using QIA shredder and RNeasy Mini Kit (Qiagen Inc., Valencia, CA, USA) according to the manufacturer’s instructions. TaqMan^®^ Gene Expression Assays (Applied Biosystems, Foster City, CA, USA; Thermo Fisher Scientific, Inc. Waltham, MA, USA) were used to design TaqMan probes for target mRNAs (CHT1, OCT1-2, CTL1-5, and the housekeeping gene β-actin) based on their mouse mRNA sequences. Accession numbers for the target gene and Assay IDs for the TaqMan probes are shown in Table 1. Data from one-step real-time PCR performed with the TaqMan RNA-to-CT 1-Step Kit (Applied Biosystems) were analyzed using the Light Cycler 96 System (Roche Diagnostics, Mannheim, Germany). Relative mRNA expression levels of target genes were calculated using the comparative cycle time method, and target gene expression was calculated relative to β-actin.

### 4.4. Immunoblotting

The subcellular localization of CTL1 and CTL2 proteins in SIM-A9 cells was performed using immunocytochemistry with reference to previous publications [17,42,43]. SIM-A9 cells were washed with Dulbecco’s Phosphate Buffered Saline (D-PBS) (Wako), lysed in RIPA buffer (Santa Cruz Biotechnology, Inc., Dallas, TX, USA) including 1 mM ethylenediaminetetraacetic acid and a protease inhibitor cocktail on ice, and centrifuged at 14,000× *g* for 15 min at 4 °C. The resulting supernatant was incubated in an equal volume of Tris-SDS β-ME sample solution (Cosmo Bio Corporation, Tokyo, Japan) at 100 °C for 5 min, and electrophoresed on a 10% Mini-PROTEAN^®^ TGX™ polyacrylamide gel (Bio-Rad Laboratories, Hercules, CA, USA) with molecular weight standards (DynaMarker Protein MultiColor III, BioDynamics Laboratory Inc., Tokyo, Japan). Proteins separated on 10% SDS-PAGE were transferred to polyvinylidene difluoride membranes (Bio-Rad Laboratories) using the Trans-Blot^®^ Turbo™ Transfer System (Bio-Rad Laboratories). The membrane was blocked with iBind^TM^ Flex Solution (Thermo Fisher Scientific, Inc.) overnight at 4 °C. Membranes were then replaced with new iBind^TM^ Flex Solution and incubated with 1 mg/mL rabbit anti-CTL1 polyclonal antibody (ab110767, Abcam plc, Cambridge, UK) and 0.5 mg/mL anti-CTL2 monoclonal antibody (3D11, Abnova, Taipei, Taiwan) at 4 °C overnight. Membranes were then washed in iBind^TM^ Flex Solution and incubated with 1 mg/mL horseradish peroxidase conjugated anti-rabbit (1:500 dilution, KPL074-1506, SeraCare, Milford, MA, USA) and anti-mouse IgG (1:500 dilution, KPL074-1806, SeraCare) for 1 h at room temperature. Visualization of protein bands was performed using ECL Prime Western Blotting Detection System (GE Healthcare Life Sciences, Marlborough, MA, USA), and ChemiDoc XRS Plus System (Bio-Rad Laboratories) was used to acquire luminescence images.

### 4.5. Immunofluorescence Staining

SIM-A9 cells grown on 35-mm glass-bottom dishes (IWAKI, Tokyo, Japan) were washed twice with D-PBS without Ca^2+^ and Mg^2+^ (Wako) and fixed with 100% methanol for 20 min at room temperature. Fixed cells were washed twice with D-PBS without Ca^2+^ and Mg^2+^, and incubated with iBind^TM^ Flex Solution overnight at 4 °C. Cells were incubated with 2 mg/mL rabbit anti-CTL1 polyclonal antibody (ab110767, Abcam plc, Cambridge, UK) in new iBind^TM^ Flex Solution overnight at 4 °C. The colocalization of CTL2 with mitochondria and Golgi was confirmed using the respective organelle marker antibodies. Primary antibody reactions for the following antibodies were performed for 4 h at room temperature: (I) anti-CTL2 mouse monoclonal antibody (4 mg/mL, 3D11, Abnova), (II) anti-COX IV polyclonal antibody (3 mg/mL, ab16056, Abcam plc), (III) anti-MG130 polyclonal antibody (1:250, PM061, Medical Biological Laboratories Co., Ltd., Nagoya, Japan). After washing with iBind^TM^ Flex Solution, the cells were incubated with 2 mg/mL Alexa Fluor 488 goat anti-rabbit IgG (Molecular Probes Inc., Eugene, OR, USA), 2 mg/mL Alexa Fluor 488 goat anti-mouse IgG (Molecular Probes Inc.), and 2 mg/mL Alexa Fluor 568 goat anti rabbit IgG (Molecular Probes Inc.) for 1 h at room temperature. After washing out the excess antibody with iBind^TM^ Flex Solution, the specimens were mounted using VECTASHIELD mounting medium with 4′, 6-diamidino-2-phenylindole (DAPI) (Vector Laboratories, Inc., Burlingame, CA, USA). Fluorescence images were obtained using a confocal laser scanning biological microscope (FV10i-DOC, Olympus, Japan).

### 4.6. Analysis of [^3^H]Choline Uptake into SIM-A9 Cells

[^3^H]Choline uptake analysis was performed using [^3^H]choline (specific activity: 2800 GBq/mmol, PerkinElmer Life Sciences, Inc.), with reference to previous reports [17,18]. SIM-A9 cells were cultured in noncoated 24-well culture plates. Cells were washed twice with uptake buffer (125 mM NaCl, 4.8 mM KCl, 1.2 mM CaCl_2_, 1.2 mM KH_2_PO_4_, 5.6 mM glucose, 1.2 mM MgSO_4_, and 25 mM 4-(2-Hydroxyethyl) piperazine-1-ethanesulfonic acid adjusted to pH 7.4 with Tris). Choline uptake was initiated by adding [^3^H]choline (PerkinElmer Life Sciences, Inc.) in a 37 °C incubator. The uptake buffer was removed and washed rapidly three times with ice-cold uptake buffer to terminate [^3^H]choline uptake. The radioactivity of aliquots of cells dissolved in 0.1 M NaOH, 0.1% Triton X-100, and liquid scintillation cocktail Hionic-Fluor (PerkinElmer Life Sciences, Inc.) was measured with a liquid scintillation counter (Tri-Carb^®^ 2100 TR, Packard Instrument Company, Meriden, CT, USA).

Na^+^-free buffer was prepared by replacing NaCl with an equimolar concentration of NMDG-Cl. Choline uptake into SIM-A9 cells was also analyzed under conditions of different pH (pH 6.0, 6.5, 7.0, 7.5, 8.0, and 8.5) by mixing 25 mM 2-Morpholinoethanesulfonic acid uptake buffer (pH 6.0) and 25 mM Tris uptake buffer (pH 8.5). Both buffers also contained 125 mM NaCl, 4.8 mM KCl, 1.2 mM CaCl_2_, 1.2 mM KH_2_PO_4_, 5.6 mM glucose, and 1.2 mM MgSO_4_. The concentration of [^3^H]choline was kept constant at 10 nM, and unlabeled choline was added at various concentrations to analyze the saturation kinetics. The difference in total [^3^H]choline uptake between the presence and absence of 30 mM unlabeled choline was defined as the specific uptake of [^3^H]choline.

### 4.7. Data Analysis

All data are presented as the mean ± standard deviation (SD). Statistical analyses were performed using GraphPad Prism 9 software (GraphPad, San Diego, CA, USA) with one-way ANOVA followed by Dunnett’s multiple comparison test, two-way ANOVA followed by Šídák’s multiple comparisons test, and the unpaired *t*-test. A *p*-value of less than 0.05 was considered to indicate a statistically significant difference. The kinetic parameters *K_m_* and *V_max_* were calculated by nonlinear regression of the Michaelis–Menten equation and was confirmed by linear regression of the Eadie–Hofstee plot using GraphPad Prism 9.

## Figures and Tables

**Table 1:** TaqMan probes used in the gene expression assay.

Target Gene	Accession Number	Assay ID
CTL1 (slc44a1)	NM_001159633.1	Mm01350815_m1
CTL2 (slc44a2)	NM_001199186.1	Mm00507664_m1
CTL3 (slc44a3)	NM_145394.3	Mm00520420_m1
CTL4 (slc44a4)	NM_023557.3	Mm00469893_m1
CTL5 (slc44a5)	NM_001081263.1	Mm01317372_m1
CHT1 (slc5a7)	NM_022025.4	Mm00452075_m1
OCT1 (slc22a1)	NM_009202.5	Mm00456303_m1
OCT2 (slc22a2)	NM_013667.2	Mm00457295_m1
β-actin	AK078935.1	Mm00607939_s1

## Data Availability

Materials described in the manuscript, including all relevant raw data, will be freely available to any scientist wishing to use them for non-commercial purposes upon request via e-mail to the corresponding author (M.I.).
